# Behavioral Measurement as Centerpiece

**DOI:** 10.1007/s40614-024-00429-x

**Published:** 2024-12-19

**Authors:** James M. Johnston

**Affiliations:** Knoxville, TN USA

**Keywords:** Pennypacker, Behavioral measurement, Applied behavior analysis

## Abstract

Hank Pennypacker’s interest in behavioral measurement began early in his career and gradually became the centerpiece of his diverse accomplishments. A review of his focus in the 1960s is followed by a brief summary of the evolution of behavioral measurement practices in applied behavior analysis as it emerged from the field’s laboratory history. This examination serves as pretext for a discussion of the contributions of *Strategies and Tactics of Human Behavioral Research* (Johnston & Pennypacker, 1980), *Strategies and Tactics of Behavioral Research *(Johnston & Pennypacker, 1993a, 2009), and *Strategies and Tactics of Behavioral Research and Practice (*Johnston et al., 2020) to emerging matters of behavioral measurement. That discussion focuses on how issues raised by the challenges of applied research and practice have evolved.

Henry S. Pennypacker—always “Hank” to all who knew him—joined the faculty in the Department of Psychology at the University of Florida in 1962, having recently completed his doctorate under Gregory Kimble at Duke University. His initial focus on classical conditioning of the eyelid reflex in monkeys gave him limited preparation for the career he was to build at the University of Florida.

The transition in Hank’s interests from classical conditioning to behavior analysis was most noticeable in 1968. In the fall of that year, Ed Malagodi joined him on the faculty, bringing graduate training in behavior analysis and funds to establish a research laboratory. During his inaugural year, Ed taught the program’s first graduate courses in the experimental, methodological, and conceptual literatures defining the field at the time. The selections were limited compared to today’s options, comprised mainly of readings in Honig (1966), Sidman (1960), and Skinner (1953), as well as articles in the *Journal of the Experimental Analysis of Behavior* (*JEAB*) and the first issue or two in the *Journal of Applied Behavior Analysis* (*JABA)*. This content encouraged Hank and I to share many hours of discussion during my graduate school years.

In that seminal year, Hank’s emerging focus on measurement began with what might today seem no more than trivial entertainment. The field was then morphing from a largely animal lab research enterprise toward a more expansive agenda involving applied projects tentatively demonstrating meaningful behavior change in everyday people and environments. That growing interest led to fascination with the discovery that merely measuring ordinary behaviors could be revealing and even useful. Among the curious and adventuresome, it was not uncommon to strap on a modified golf counter or even a leather wrist cuff with abacus beads and throughout the day select some almost random behaviors to count, whether for a few minutes or even all day. Hank could be caught wearing more than one counter on his wrist, and, when he felt mischievous, he would count someone’s behavior in a way they might notice.

It may seem odd, but it was quietly exciting to realize that such a simple task could be so revealing, even if nothing more came from a period of observation than the singular fact of a total count in some sampling period. That result was only a division problem away from getting a rate, thereby touching base with years of basic research. Such elementary revelations may have been of uncertain value, but were perhaps no less exciting than those made with early microscopes. At the least, there was now a fact that did not exist before, but Hank—and many others—foresaw a method with limitless potential.

In the spring of 1969, Hank invited Ogden Lindsley to conduct a 3-day trainer’s workshop in precision teaching. Lindsley’s focus on carefully measuring the behavior of individuals and displaying those data in a way that focused on practical behavior change objectives had a powerful impact on Hank’s developing interests in behavior analysis. It was precision teaching’s focus on behavioral measurement that led Hank to develop a lifelong leadership role in the precision teaching community, exemplified by his co-authorship of the *Handbook of the Standard Behavior Chart* (Pennypacker et al., 1972).

It was also during 1968 that Hank announced that we should practice in the classroom what we preached, so we embarked on an energetic program to redesign his stock undergraduate course in behavior analysis to reflect what we characterized as a behavioral approach to college teaching. The program involved assigning students to other more advanced students (called managers) who were experts in the subject matter of the course. After attending lectures and reading the text, students scheduled a meeting with their manager to answer fill-in questions presented in a flip-card format. That timed performance was recorded and displayed on the student’s graph in terms of rates of correct and incorrect responding relative to the course’s stated criterion. That record of the student’s verbal behavior was then the basis for individual instruction by the manager. Performances falling short of minimum rate criteria required students to schedule additional performance sessions until criterion performance was achieved. (Johnston & Pennypacker, 1972). The foundation of the course was repeated measurement of each student’s verbal performances addressing each unit of course material, supplemented by maintenance of a graphical record throughout the term.

It was only a few years later that Hank began a robust research program concerning breast self-examination (see Pilgrim, forthcoming). Although there were many methodological facets of this research effort, measurement in different forms was always its guiding focus, from measuring the hardness of cancerous tissue retrieved from surgery to the psychophysics of manual palpation of artificial breast tissue to detect embedded lumps. This federally funded initiative eventually evolved into a masterful entrepreneurial demonstration of how to transfer behavioral technology into the marketplace (Pennypacker, 1986).

## A Growing Need to Understand Behavioral Measurement

In retrospect, it is easy to trace the development of Hank’s career-long focus on the role of measurement in behavior analysis. That interest is best represented in our book, *Strategies and Tactics of Behavioral Research and Practice* (S&T; Johnston et al., 2020), originally published with the title, *Strategies and Tactics of Human Behavioral Research* (Johnston & Pennypacker, 1980). The remainder of this article is an informal summary of evolving measurement practices in the growing applied community and how they were addressed in successive editions of S&T.

Until our book was published, the sole treatment of the research methods characterizing the field’s history was Murray Sidman’s (1960) text, *Tactics of Scientific Research*. His seminal volume described the methods developed by Skinner and others that were the basis for the effectiveness of early animal laboratory investigations of operant learning processes. Although Sidman thoroughly explained the procedures and reasoning guiding the methods by which experimental data were analyzed, he included almost no discussion of how they were collected. That omission was understandable because research settings at that time were usually laboratory preparations in which responses were typically key pecks, lever presses, and other simple, brief actions. These were automatically transduced by laboratory equipment, with timing similarly recorded. The most common dimensional focus was count, which usually served to calculate rate of responding, often displayed graphically in automatically drawn cumulative records. Skinner noted in an early interview that the measurement of rate of responding and its cumulative display was his most significant contribution (Evans, 1968).

As research interests throughout the 1960s increasingly extended to human subjects behaving in nonlaboratory settings, the methodological demands of behavioral measurement grew more complicated. For example, target behaviors were rarely so simple as a key peck or a lever press, so definitional practices in field studies warranted attention that was not customarily forthcoming. The challenges of selecting targets that suited the needs of the experimental question, the requirements of treatment conditions, and the risks of influences unrelated to treatment variables were new to applied projects. The tendency was to describe target behaviors in what seemed to be obvious everyday terms. The primary consideration underlying definitions seemed to be guiding observer recording assignments and framing conclusions about experimental outcomes in terms of researcher interests and the apparent needs of the literature. Matters of topographical versus functional characteristics were not common considerations, in spite of Skinner’s earlier treatments (Skinner, 1935, 1938, 1945). One outcome of this approach was growing areas of literature referenced by broad behavioral labels (e.g., aggressive behavior, play behavior) that implied a degree of generality that was not necessarily appropriate, or at least not empirically substantiated.

In these early years, the laboratory simplicity of automatically counting and timing continuous samples of the target behavior was rapidly overwhelmed by burgeoning applied interests. Human observers became the default tansducers in the face of endless variety in experimental questions, settings, target behaviors, and observational scenarios. This in turn shifted observation interests toward the selection of sampling periods defining sessions and sampling rules within sessions. As a result, the formalities of dimensional measurement in applied projects faded in importance, and other influences encouraged a broader array of options. For example, developmental psychology had a notable impact on observational procedures in field settings, as described by Hartman (1978). The editorial leadership of the new *Journal of Applied Behavior Analysis* seemed comfortable with the ascendence of what is now called interval recording, as did most authors and reviewers.

A related aspect of behavioral observation involves decisions about how many sessions are necessary. In animal laboratory research preparations discussed by Sidman (1960), achieving stable responding across multiple sessions under each condition was typically a high priority, and relatively lengthy phases were the norm. In contrast, accumulating multiple successive sessions in each condition under applied circumstances was understandably more challenging, and as field research began to dominate the literature, the number of sessions comprising typical phases grew much shorter. In fact, the modal number of sessions in each phase in *JABA* studies from 1968 through 1977 was between three and four (Huitema, 1986). The price of declining respect for the role of steady states in experimental projects cannot be determined due to a lack of evidence about the effects of more exposures to each condition.

The final task of behavioral measurement is to assess the quality of the data as they are being collected and certainly before drawing conclusions. In laboratory settings, this usually involved making sure that target responses were correctly transduced by the equipment as an experiment progressed. Records of this assessment were not usually retained because any malfunctions were fixed on the spot, misleading data were thrown out, and the luxury of long phases made it easy to simply gather more data. As observers replaced equipment, assessing their performance typically involved occasionally comparing their data to data concurrently collected by other observers. The calculated comparison of the data from primary and secondary observers was called interobserver agreement (IOA), and this practice remains routine today. This approach leaves questions about accuracy and reliability, which are not accessible with IOA data, unanswered.

In sum, the welcome growth of applied research, beginning in the 1960s, embodied a transition in the methods of behavioral measurement that had been well-established in the animal laboratory. Sidman’s otherwise painstaking discussion of the young field’s analytical methods in 1960 understandably failed to anticipate some of the methodological needs that would later be revealed by a new applied journal (*JABA*) and the literature it would encourage. The challenges raised by certain measurement procedures seemed to pass unrecognized and often gradually became routine practice.

## A New Methods Text

The previous section summarizes what behavioral measurement looked like when Hank and I started talking about writing a book on research methods in the early 1970s. We were guided by Sidman’s volume when it came to analysis but learned that we were woefully ignorant about many aspects of behavioral measurement. The organization of S&T’s content into *strategic* objectives versus *tactical* options helped disentangle overarching issues from practical maneuvers, including details that might be considered neither strategies nor tactics, but techniques. We revised the definition of behavior in each edition, wrestled with terms such as frequency and rate, and steadfastly resisted the growing tendency in the culture to treat data as singular, among other struggles. We were at least clear in our objective to address what we saw as the needs of the emerging applied research literature. As applied journals proliferated, the growing literature became too substantial to master, although the roster of methodological problems it increasingly revealed became more well-defined. That helped us sharpen our treatment of particular aspects of measurement, as summarized in the following sections.

## Behavior as a Subject Matter

From the outset, the nature of behavior as a scientific subject matter served more as background than foreground in applied discussions of behavioral measurement. Although Skinner had largely rid the field of the most egregious forms of mentalism, at least in its research publications, formal definitions of behavior seem to provide limited guidance for the task of selecting target behaviors and developing comprehensive measurement protocols. Nevertheless, the implications for measurement procedures of what remains when mentalism is exorcized brings some clarity to those tasks.

The challenge of distinguishing between behavioral and biological aspects of the organism, the repercussions of the intraorganism nature of behavior, and the role of the environment in conceptions of behavior were among the topics that received only inconsistent attention in the growing applied community. We proposed that the requirements for investigating behavior as a natural phenomenon serve as the touchstone for measurement methods, thereby providing those necessary procedural details with a proper foundation. This approach was an attempt to encourage investigators to fully respect the basic characteristics of behavior, a topic that was especially important to Hank.

A variety of topics not consistently at the forefront of measurement considerations emerge from that focus. Pointing out the implications of behavior as the interface between organism and environment, warning against the slippery slope of colloquial terms hiding mentalistic assumptions, cautioning against interest in apparent behavior that does not actually exist, and clarifying the hazards of referring to group behavior are among topics that have consequences for the development of measurement procedures. This interest in group behavior was not uncommon and seemed motivated by a justifiable desire to find interventions that were broadly effective across members of a group. This not infrequently led to collating observations of multiple participants into measures of group reactions to intervention conditions. Of course, such data hide the actual effects on individual participants that might encourage a search for different sources of control among participants. The growing applied literature seemed that it might benefit from attention to all of these matters.

## Selecting Target Behaviors

The distinction between functional and topographical behavioral characteristics in defining target behaviors is a key foundation for discussion of practical issues. The understandable default tendency of selecting and defining target behaviors in early applied projects instead seemed to give priority to the practical features of target behaviors under the real-world circumstances that motivated a research initiative. That primacy risked accommodating some of the misdirection associated with common parlance, as well as leading to unrelated influences embedded in everyday environments that could be confused with the effects of treatment variables.

Avoiding such problems requires a systematic and detailed consideration of each of the roles a target behavior must play in a project, which requires identifying the specific characteristics of a target behavior necessary for serving each role. For example, we emphasized that selected behaviors must be relevant to the intervention condition so that findings are practically meaningful. They must also be sensitive to any possible effects of the intervention condition, a requirement that means that it must be defined neither too broadly nor too narrowly. Furthermore, they must be able to vary in ways that will reveal treatment effects.

We argued that accommodating these factors requires a thorough evaluation of the characteristics of prospective target behaviors to determine whether certain features might cloud the clarity with which the effects of a treatment condition are revealed. How might certain features of the behavior change when exposed to experimental conditions? Would certain aspects of a behavior tend to encourage an increase or a decrease in responding? Would the behavior be able to vary in either direction or be stymied by a floor or ceiling of some sort? What extraneous variables to which the behavior could be sensitive might bias or even overwhelm treatment effects that might otherwise be evident?

It is unavoidable that the circumstances under which applied research is conducted often provide constraints on the investigator’s options in selecting and defining target behaviors. The choices are typically tied to the practical nature of a larger behavioral issue and particular environmental circumstances defining each project. This can make it difficult for investigators to select and define target behaviors that accommodate the needs of all of the other components of a sound study. Nevertheless, this challenge does not weaken the importance of decisions about target behaviors and how they might reveal treatment effects.

## Dimensional Measurement

Sound behavioral measurement describes the target behavior in terms of at least one of its physical aspects. For many years, the default dimensions depicted in laboratory data were count and rate. Throughout the history of applied research, these dimensions also remained popular choices. Measures of duration were common as well, and, perhaps less often, some form of latency was selected for observation. Interresponse times, celeration, and topographical dimensions were not as often measured.

Until S&T was published, there was little discussion of the possible reasons for choosing one dimension over another or even how these dimensions might be defined and calculated. For example, there are different ways of calculating rate of responding that can lead to importantly different outcomes. Other issues, such as why multiple dimensions might be concurrently measured, the limitations of ratios, the risks of dimensionless ratios, and constraints on how dimensional data might be described had yet to gain much attention. (See chapter 5 of Johnston et al., 2020, for discussion of these topics.)

Our discussion of dimensional measurement unavoidably confronted the conflict between that approach and the use of interval-based measurement procedures. These procedures have been widely used in the applied literature since the early years of *JABA*, are routinely described and recommended in textbooks, and show little sign of falling from favor. Nevertheless, we pointed out that interval-based procedures are notable for their shortcomings, as detailed in a solid empirical literature. (These studies are listed and discussed in chapter 6 of Johnston et al., 2020). Although these interval-based procedures can be faulted on multiple grounds, we noted that their fundamental failing is that they do not directly reflect actual dimensions of behavior. The studies documenting and explaining the problems with interval-based observation rules show how such data can paint pictures of apparent changes in behavior that can be misleading. Furthermore, as our discussion illustrates, merely changing the duration of observation intervals is likely to change the interval scoring outcomes for any given data set, which can substantially change the data to be analyzed.

## Observation

The key task of collecting behavioral data presented the growing applied research agenda with a number of challenges. Various constraints associated with applied interests sometimes made it difficult to directly measure the actual target behavior, usually the focus of conclusions. Such circumstances encouraged measuring more easily accessed behaviors and using those data as a basis for inferences about the impact of experimental conditions on the real behavior of interest, a practice we defined as indirect measurement. We noted that direct measurement unquestionably has the advantage because indirect measurement carries the burden of determining how well the resulting data reflect what actually happened with the behavior, an obligation that is usually challenging and therefore frequently ignored.

Deciding when observation should occur requires yet another set of considerations. In attempting to highlight these issues, S&T made a distinction between complete and incomplete measurement. The former involves scheduling observations that will capture all occurrences of the behavior. The latter approach schedules periods of observation that only sample from all opportunities for responding. Although measuring behavior as often and as long as possible is ideal, it is not clear that this priority has dominated sampling decisions in the applied literature over the years. The circumstances under which the target behavior naturally occurs must be taken into account, perhaps balanced by the desirability and challenges of creating a more controlled research environment that would offer preferable sampling opportunities. The availability of observers, not to mention their ability to perform consistently throughout a sampling period, is also an understandable concern. Our discussion of sampling issues acknowledged that compromises between better and worse alternatives are often required, in spite of the risk of undesirable consequences.

## Evaluating Collected Data

The general approach to evaluating the results of measurement procedures in the natural sciences requires assessing the accuracy and reliability of data. In studies that collect data using automated equipment, this may require little more than ensuring that the equipment is operating as desired. Developing automated options might often be seen as an unnecessary digression from more important applied interests, however, which has long encouraged the use of human observers. The general approach throughout the evolution of applied research has been to select individuals who may have experience that benefits their performance as observers, provide them with task specific training, and monitor their compliance with observation protocols. Evaluating the resulting data has usually been accomplished by comparing the data collected by primary observers to data periodically collected by secondary observers—assignments that are often arbitrary. Interobserver agreement (IOA) data do not provide the means for assessing accuracy or reliability.

The literature that has accumulated concerning the possible mechanisms associated with IOA addresses matters such as number and timing of IOA data collection efforts, ways of comparing primary and secondary observer data, standards for IOA outcomes, and formats for reporting IOA comparisons. Given the limitations of IOA data, S&T introduced the term *believability* to characterize the objective of IOA procedures—that is, encouraging users to believe that the data collected by the primary observer are good enough to accept for purposes of analyzing experimental outcomes and drawing conclusions.

The considerable procedural variations in the collection of IOA data make it difficult to interpret agreement outcomes from one study to another. In any case, there is no universally agreed upon standard for the degree of agreement that justifies accepting primary observer data as acceptable for analysis (Cooper et al., 2007; Kazdin, 2021). However, it is not uncommon for a minimum of 80% agreement to be considered satisfactory, a standard often suggested in published studies and recommended in some textbooks (e.g., Bailey & Burch, 2002; Cooper et al., 2007; Kennedy, 2005). Because the implications of 80% agreement for any particular comparison of primary and secondary data sets cannot be fully and unambiguously known, this begs the question of how well the primary data set in a study represents the actual behavior of participants.

## Progress, or Lack Thereof

The previous section summarizes the behavioral measurement issues that emerged with the development of the applied literature over the past 60 years or so. S&T addressed these issues with increasing thoroughness over four editions, attempting to more fully accommodate the field’s needs.

It is difficult to assess the contribution of S&T to the measurement practices that characterize the applied research literature and what is now a very large service delivery community. It is at least clear that some of the book’s recommendations and arguments have made an impression because they are not infrequently represented in other textbooks (e.g., Kennedy, 2005). On the other hand, it is no less clear from published articles that some troubling measurement practices, briefly described above, remain relatively unscathed.

In sum, Hank Pennypacker’s focus on behavioral measurement was not only the centerpiece of his career but of the field’s approach to research and service delivery. Over the years, he helped identify and explain its many facets, often exemplifying best practices in his own research. From the outset, he understood that the credibility of the literature’s findings, the effectiveness of its technologies, and the directions suggested for future research and practical development unavoidably depend on the quality of the field’s measurement practices. Indeed, the credibility and reputation of behavior analysis come down to how we measure behavior.
